# Round the Hospitals

**Published:** 1919-07-12

**Authors:** 


### ROUND THE HOSPITALS.

The Queen has taken the occasion of the winding up of the orthopædic section of her Needlework Guild, the last to close down, to send a letter of appreciation to the committee of the Surgical Requisites Association. Her Majesty expresses her "great appreciation of the wonderful ingenuity of individual workers, as well as of the great self-sacrifice of all concerned, all over the country." It has indeed been an object-lesson to the country to watch the spirit of joy with which pleasure-loving women laid aside their toys at the call of the wounded. If only the impulse for service can be kept alive now peace has come, what may not be accomplished in the days ahead?

When the adjourned debate on the Nurses' Registration Bill was resumed in the House of Commons on July 4, Major Nall stated that he had received a letter from the College of Nursing, announcing their willingness to withdraw their Bill in the

**ROUND THE HOSPITALS—(*continued*).**

House of Lords on the Government's undertaking to introduce a Registration Bill. The House assented to his motion that further consideration of the Central Committee's Bill be adjourned *sine die*, and that page in the history of State Registration is therefore turned down. There can be no doubt that the interests to be considered are too many and powerful for any private Bill to command assent from the whole profession. The delay in dealing with this most important national matter, since it ensures a thorough examination into the root causes of the opposition offered by the College to the Central Committee's Bill, is all for the good.

A TOUCHING memento of Edith Cavell has come into the possession of the Star and Garter Hospital at Richmond. It consists of two tiny scraps of grey notepaper on which Nurse Cavell recorded the events of her last hours. There is no sign of discomposure in the small neat writing. Each stage of the events preceding her execution is noted down as though she sat at her usual desk filling in her diary for the day. To stand before the little frame in which these priceless fragments are preserved is to reach some faint conception of the unruffled calm which sustained her in the hour of death.

THE annual meeting of the Royal British Nurses' Association can hardly fail to have produced a sense of deep discouragement in those who used to love the Corporation and have stood by it through many difficulties. Six societies, the total membership of which was not stated, have affiliated to the Chartered Corporation, and secure the privilege of nominating each a representative for election to the General Council. It is not stated whether the members of these societies become registered members of the Royal British Nurses' Association by this act, or whether all these members are qualified for registration, but it is certainly startling to find the entire Fever Nurses' Association taken over *en bloc* by a Corporation which has always stood for a three-years' general training. The registration fee has been lowered from a guinea to five shillings, "in order that more nurses might be able to make use of the charter." The success of this policy may be gauged by the number of nurses who presented themselves for registration during the year. They totalled 148, of whom 141 were accepted. Membership subscriptions amounted to £110, which, even supposing none were life subscriptions of £2 2*s*., would give a subscribing membership of 440.

IF these figures of the Royal British Nurses' Association and the six societies affiliated to it be contrasted by puzzled members of Parliament with the ever-increasing membership of the College of Nursing, now well on into its fifteenth thousand, all paying a guinea for the privilege of membership and registration, they will find themselves faced with a choice between two alternatives. Either the nursing profession whole-heartedly and freely endorses the policy of the College of Nursing and accepts its representatives as their representatives, or it stands revealed as the most extraordinarily whimsical and self-contradictory body of women the world has ever seen. If all the people who joined the College were probationers in training it might be possible to imagine their matrons terrorised them. But as a matter of fact nurses are not eligible for acceptance by the College until they have received their certificates and are independent of the matron's control.

THE British Committee of the Russian Red Cross is appealing for nurses and V.A.D. uniforms for nurses and sisters in Russia who are stated to be completely destitute, and unable to procure any clothing in that country. Any good cast-off articles of attire would be useful, and may be sent to the headquarters of the British Committee, Russian Red Cross, 35 Albemarle Street, W. 1. There is an urgent call to assist the sick and wounded in Russia, where people are dying in tens of thousands of typhoid, cholera, and scurvy. In Petrograd the epidemics have reached incredible dimensions; the sick, finding no refuge, wander about the streets infecting others, and die on the pavements or in some courtyard where they have crawled out of the way. The doctors, nurses, and assistants are foredone with work and privations. At Ekaterinodar 50 per cent. of the nurses are ill with typhoid. Ghastly famine, beyond the horrors of a siege, completes the universal misery.

A WHITE Paper has been issued, in which Sir Edward Ward attempts to give some account of the work put through by the 400,000 people who made comforts for the sick and wounded under the Army Council's scheme during the war. The articles of clothing and surgical comforts turned out mounted to the respectable total of eighty-eight million, and "the noble self-sacrifice of the great band of workers at home" finds fitting acknowledgment from the Director-General.

THE Thanksgiving Services in town and country last Sunday were almost stupifying to some minds in the weight of memories they evoked. No more admirable service was ever put forth by the Archbishops for congregational use. The reflection came irresistibly to the mind in watching the effect produced on crowds of worshippers, not always to be seen in church, how finely might public worship be transformed were a corresponding effort habitually made to interpret the minds of ordinary men and women to themselves. Thanksgiving is less easy than supplication. The act of gratitude is among the highest to which human nature can aspire. But on Sunday all were attuned to this high note, by a service which owed none of its solemnity to musical grandeur, and even unsung would have been impressive. In nearly all institutions for the sick a poignant element was introduced by the presence of some at least of the wounded, the

**ROUND THE HOSPITALS—(*continued*).**

overstrained, the disabled, the blinded, the bereaved, whose sufferings were remembered in the nation's act of praise.

---

The magnitude of the tasks waiting to be accomplished by nurses in the Empire is strikingly illustrated in the Report of the Overseas Nursing Association. In 1919 the number of nurses at work is seventy-five, a rapid recovery from the war years, when owing to the lack of steamship accommodation many posts perforce remained long vacant. The largest number at work in the history of the association was ninety-six in 1914, and it is probable that this number will be largely exceeded before very long. The need for maternity nurses in the isolated districts of the Dominions is overwhelming. It seems that over 35,000 British women have married Canadian soldiers, and that marriages are taking place at the rate of 1,000 a month. The return of these couples to Canada calls for a very special effort to provide maternity aid, since in Saskatchewan alone it was estimated in 1914 that one woman in three was materially injured in childbirth. A special Dominion Fund is being raised for this purpose. The best solution of the problem would be to turn some of the settlers themselves into midwives, so that they might help each other. More facilities for midwifery training are required both in this country and Canada if the necessary aid is to be forthcoming for the settlers.

---

Influenza plague and cholera have proved terrible obstacles to the advance of mission work in India during the past year. The report of the Zenana Bible and Medical Mission for 1918 bears witness to the ravages in particular of the first-named disease, which last autumn came like a terrific storm, attacking all sorts and conditions of men, women, and children. From every district of India come painful reports of mission-hospitals closed altogether or crippled in usefulness for want of an adequate staff. At Lucknow, as elsewhere, the mission work is intensely appreciated, but more women doctors and fully qualified nurses are indispensable to further progress. A movement is on foot for raising the sum of £25,000 to consolidate and extend the work of the Zenana Mission among the women and children of India by raising the allowance of all missionaries (at present it is but £120 a year), by re-opening institutions closed down during the war, extending the schools and educational work generally, and increasing the accommodation for orphan and famine children.

---

Miss Mabel Bootman, probationer at the Bermondsey Infirmary, has successfully appealed against the fine of £2 imposed on her by Mr. Gill for being concerned in the assault against a fellow-probationer. Miss Bootman gave evidence denying that she had taken any part in the "ragging" incident, and declared that she knew nothing about the plan to duck Miss Russell in the bath. The appeal was allowed, and though we have abstained from publishing the names of the delinquents we are glad to lend publicity to the disavowal.

---

The department for massage and physical and remedial exercises at St. Thomas's is very popular with members of Voluntary Aid Detachments, many of whom are now in training there for appointments. The neat brown uniform worn by the students is a familiar feature in the wards, where much of the massage is done by them under the superintendence of their teachers. The advantages they enjoy will shortly be extended by the opening of a new electrical department. The career is very suitable for women who find secretarial and other sedentary employments injurious to their health. Its effects in stimulating the vitality of the learners and banishing ailments is quite remarkable.

---

Some interesting features of infant mortality were brought to note at the last meeting of the National Birth-rate Commission. Dr. Eric Pritchard stated in his evidence that infection of one kind or another was responsible for much the largest number of the deaths that occurred during the first year of life. Good mothercraft was knowingly or unknowingly designed to preserve the natural defences of the body and stimulate the infants' resistance to enemy organisms. Mothers in every class of society need to have this truth brought forcibly before them. It helps to explain why the process of excluding every possible source of risk as practised in rich women's nurseries is less conducive to sturdy childhood than that unconsciously practised by the poor, who succeed in raising the degree of tissue immunity in their babies by the natural means of breast-feeding, fresh air, and absence of fussing.

---

It is not perhaps as widely known by nurses as it ought to be that loans are procurable from the Central Bureau Loan Fund, 5 Prince's Street, Cavendish Square, W. 1, to enable them to take public health, midwifery, or massage training. Applicants are expected to bring satisfactory testimony as to their suitability for the chosen career, and to furnish two guarantors, who are responsible householders, to ensure the repayment of the loan by monthly or quarterly instalments. Last year 123 women received loans in order to fit themselves for their career, of whom five received training in public health work, thirteen in domestic science, four in medicine, and four in midwifery. Nurses are often handicapped at the conclusion of their training owing to the want of ready money which would enable them to take an extra course to qualify for special posts, and the opportunity offered by this bureau is most opportune at the present time.

---

In The Hospital of November 30, 1918, and subsequent issues we dealt with the great changes and improvements which the Board of the Leeds General Infirmary had agreed to in regard to the remuneration, privileges, and pay of the nursing staff. To-day we have the pleasure to give in the

**ROUND THE HOSPITALS—(*continued*).**

succeeding paragraphs some further facts, which show the progressive character of the great improvements which the authorities are continuing to make for the benefit of the fully-qualified nursing staff, and also of the probationers at present being trained and of those yet to come. This is all to the good, but we welcome with special cordiality the increase in the salaries of the ward and theatre sisters from £60 to £85 a year, the latter sisters as a class having often most arduous duties, taxing their capacity and strength to the maximum. It is further a great matter that, following the lead of St. Thomas's Hospital, Leeds is now to have a sister-tutor, and that she will receive a commencing salary of £100 per annum. In offering our congratulations and thanks to the zealous, inspiring, hard-working, and devoted chairman, Mr. Charles Lupton, who has always shown so keen an interest in the welfare of the nursing staff, ably supported as he has been by Mr. Joseph Watson, another friend of the nurses, we must emphasise the splendid services to the General Infirmary, Leeds, which Mr. Lupton has rendered during many years. Would that every voluntary hospital, at least of importance, had such a chairman.

The step taken last year in dividing the Sussex County Nursing Association in two to meet the varying needs of the east and west parts of the county has proved very successful. The new subscriptions to the East Sussex County Nursing Federation already exceed those apportioned to East Sussex under the old arrangement. Substantial Government grants in aid of the struggling district nurses' associations have facilitated development of the Federation. The energy and ability of the county superintendent, Miss Hingeston-Radcliffe, has had a considerable share in keeping up a high nursing standard throughout the entire district, and the loyalty of the district and village nurses commands high praise.

It would seem that candidates in the West Country are more disposed to take general training than to become village or maternity nurses. The Kingswood and District Nursing Association has to deplore "a dearth of ladies willing to undergo training," and has been obliged on this account to engage fully-trained nurses to carry on the work. It is possible that the training-home lacks attractiveness or advertising enterprise, or surely some of the superfluous candidates at the Bristol General Hospital might have been lured in that direction. It is discouraging to learn that the health work under the county has been abandoned by the Kingswood Nursing Association for financial reasons. This should be looked into and remedied forthwith.

The Bristol General Hospital had its full share of the hardships entailed by the influenza outbreak last autumn, and the annual report speaks warmly of the able manner in which the matron, Miss A. E. Densham, and her depleted staff dealt with the situation. The numerous allusions to these difficulties which recur in the pages of last year's reports from institutions suggest the need for keeping afoot some link between the hospitals and the Red Cross detachments. Had there been a group of V.A.D. workers available last year who could be called on for civil service in emergencies many valuable lives would have been spared and much painful dislocation could have been avoided. The Bristol General Hospital received 160 applications for training to fill twenty-two vacancies, an encouraging sign of the popularity of the training-school.

The Royal Victorian Trained Nurses' Association in Melbourne has lately been entrusted with a delicate and difficult task by the Board of Health in the registration of partly trained nurses and V.A.D. helpers. The task is being accomplished with great ability and success. The idea is to make systematic provision in the event of epidemics such as that recently of influenza, when the trained nurses were insufficient in number to cope with the disease. The Association has recommended an increase of pay for private nurses undertaking influenza cases from £3 8s. 6d. per week to £4 4s. For semi-trained nurses the rate of pay is £2 12s. 6d. for No. 1 auxiliaries and 25s. for pantry helpers. Owing to the epidemic the proposed reforms in the conditions under which probationers are trained in the State hospitals have been postponed.

The Board of the Leeds General Infirmary, on the request of the Lady Superintendent, have decided to open a preliminary training school for intending probationers. Probationers will receive from six to eight weeks' training on the principles of nursing, and will be given preparatory lectures on elementary anatomy and physiology, hygiene, etc., and will be required to pass an examination before they enter the wards. The trial period will include the time spent in the preliminary training school, and at least one month in the wards—in all three months. Probationers in the preliminary training school will receive board, etc., and a proportion of uniform, and if they remain after the trial period on the staff of the hospital the first year's salary from the day they enter the preliminary training school. A sister-tutor is to be appointed to give instruction to the pupils in the preliminary training school. She will also give lectures on nursing to first-year nurses, and attend all lectures given by the honorary staff and coach nurses on the subjects of the lectures. The Board also, on the proposal of Mr. Joseph Watson, a member of the Board, and Mr. Charles Lupton, the chairman, agreed to pay the nurses in training as follows: first year, £20; second year, £25; third year, £30; fourth year, £40; the ward and theatre sisters, from £60 to £85 a year. The sister-tutor will receive a salary of £100. We hope to give even fuller details in a future issue, with other interesting particulars and facts.

